# Molecular insights into GPCR mechanisms for drugs of abuse

**DOI:** 10.1016/j.jbc.2023.105176

**Published:** 2023-08-18

**Authors:** Omar B. Sanchez-Reyes, Gregory Zilberg, John D. McCorvy, Daniel Wacker

**Affiliations:** 1 Department of Pharmacological Sciences, Icahn School of Medicine at Mount Sinai, New York, New York, USA; 2 Department of Neuroscience, Icahn School of Medicine at Mount Sinai, New York, New York, USA; 3 Department of Cell Biology, Neurobiology, and Anatomy, Medical College of Wisconsin, Milwaukee, Wisconsin, USA

**Keywords:** drugs of abuse, GPCR, structure, pharmacology, opioid, cannabinoid, serotonin

## Abstract

Substance abuse is on the rise, and while many people may use illicit drugs mainly due to their rewarding effects, their societal impact can range from severe, as is the case for opioids, to promising, as is the case for psychedelics. Common with all these drugs’ mechanisms of action are G protein–coupled receptors (GPCRs), which lie at the center of how these drugs mediate inebriation, lethality, and therapeutic effects. Opioids like fentanyl, cannabinoids like tetrahydrocannabinol, and psychedelics like lysergic acid diethylamide all directly bind to GPCRs to initiate signaling which elicits their physiological actions. We herein review recent structural studies and provide insights into the molecular mechanisms of opioids, cannabinoids, and psychedelics at their respective GPCR subtypes. We further discuss how such mechanistic insights facilitate drug discovery, either toward the development of novel therapies to combat drug abuse or toward harnessing therapeutic potential.

Psychoactive substances include a wide variety of both medicinal and illicit drugs that exert their pharmacological effects directly *via* modulation of the central nervous system (CNS). Many illicit drugs have become part of the daily life for a growing number of users. According to the National Institute of Drug Abuse, 17 to 41% high-school adolescents in 2022 may face a lifetime of use (1, 2). Drugs of abuse include illicit substances such as cocaine, heroin, methamphetamine, marijuana, or lysergic acid diethylamide (LSD), as well as legal drugs including alcohol and nicotine. Prescription medications such as amphetamines or opioids are also classified as drugs of abuse, and overprescription of drugs such as the opioid oxycodone (OxyContin) have played a major role in the development of an opioid epidemic. Together, consumption and abuse of these drugs greatly affect public health with severe impact on the individual, society as a whole, and the healthcare system. For instance, over a million Americans died of drug overdoses since 1999, with over 75% of the cases in 2021 involving opioids (2, 3). A commonality among drugs of abuse is their psychoactive properties, which not only cause pleasant sensations and inebriated states, but can also lead to strong dependence and drug addiction. Most of these drugs cause substantial harm to the user through potentially lethal side effects such as opioid-mediated respiratory depression.

G protein–coupled receptors (GPCRs), the largest family of membrane proteins encoded in the human genome, mediate many of the psychoactive substance effects *via* G protein–dependent or G protein–independent signaling pathways. Drugs of abuse vary greatly in their molecular targets and mechanisms of action and can display polypharmacology (ability to interact with multiple targets). Their (patho)physiology converges in the CNS, where many drugs impact GPCR signaling. For example, cocaine or amphetamines inhibit or modulate the function of transporters that mediate the reuptake of dopamine into presynaptic terminals, thereby prolonging signaling *via* dopamine GPCRs (4). However, for many classes of drugs of abuse, GPCRs are the primary molecular targets. Opioids, such as fentanyl and morphine (5), cannabinoids, such as tetrahydrocannabinol (THC) (6), and psychedelics, such as LSD and psilocybin (7), directly activate GPCRs to elicit psychoactive effects. Over the past 10+ years, a wealth of structural and pharmacological studies have elucidated how psychoactive drugs engage their target receptors. In this review, we summarize recent structural studies that uncover how the three major psychoactive drug classes (opioids, cannabinoids, and psychedelics) directly engage their respective GPCRs at the molecular level. We further summarize how molecular insights are being leveraged to design novel therapies to overcome the dichotomy of their effects.

GPCRs signal predominantly *via* G proteins but can also recruit β-arrestin and GPCR kinases to cause desensitization, internalization, and downregulation. β-arrestin potentially serves as a scaffold to regulate Src/ERK signaling (8, 9) (Fig. 1*A*), which has recently been challenged, as this appears to be GPCR- and tissue-specific (10, 11). GPCRs also exist in inactive and active states depending on ligands and/or intracellular effectors bound (Fig. 1*B*). Although GPCRs share commonalities in their activation mechanisms, recent structural studies have uncovered receptor specific features (Fig. 1*C*) that play critical roles in the activation of the distinct GPCRs. It should be noted that most GPCR structures reviewed herein contain modifications of the receptor and/or the G protein, as in the case of active-state cryo-EM structures. Nearly all antagonist-bound or inactive GPCR structures published to date have been determined using X-ray crystallography. Given that GPCRs are dynamic proteins and form ensembles of conformations, crystallization requires the inclusion of various mutations and fusion proteins to provide crystal contacts and increase both thermostability and level of expression. Likewise, active-state structures of receptor–transducer complexes determined by cryo-EM often require the use of dominant-negative G protein constructs and/or stabilizing antibody fragments to prevent dissociation from the receptor. As such, some caution must be taken in interpreting structural insights based on these different constructs as exactly equal to their wild-type unmodified native counterparts (12).

**Figure 1:**
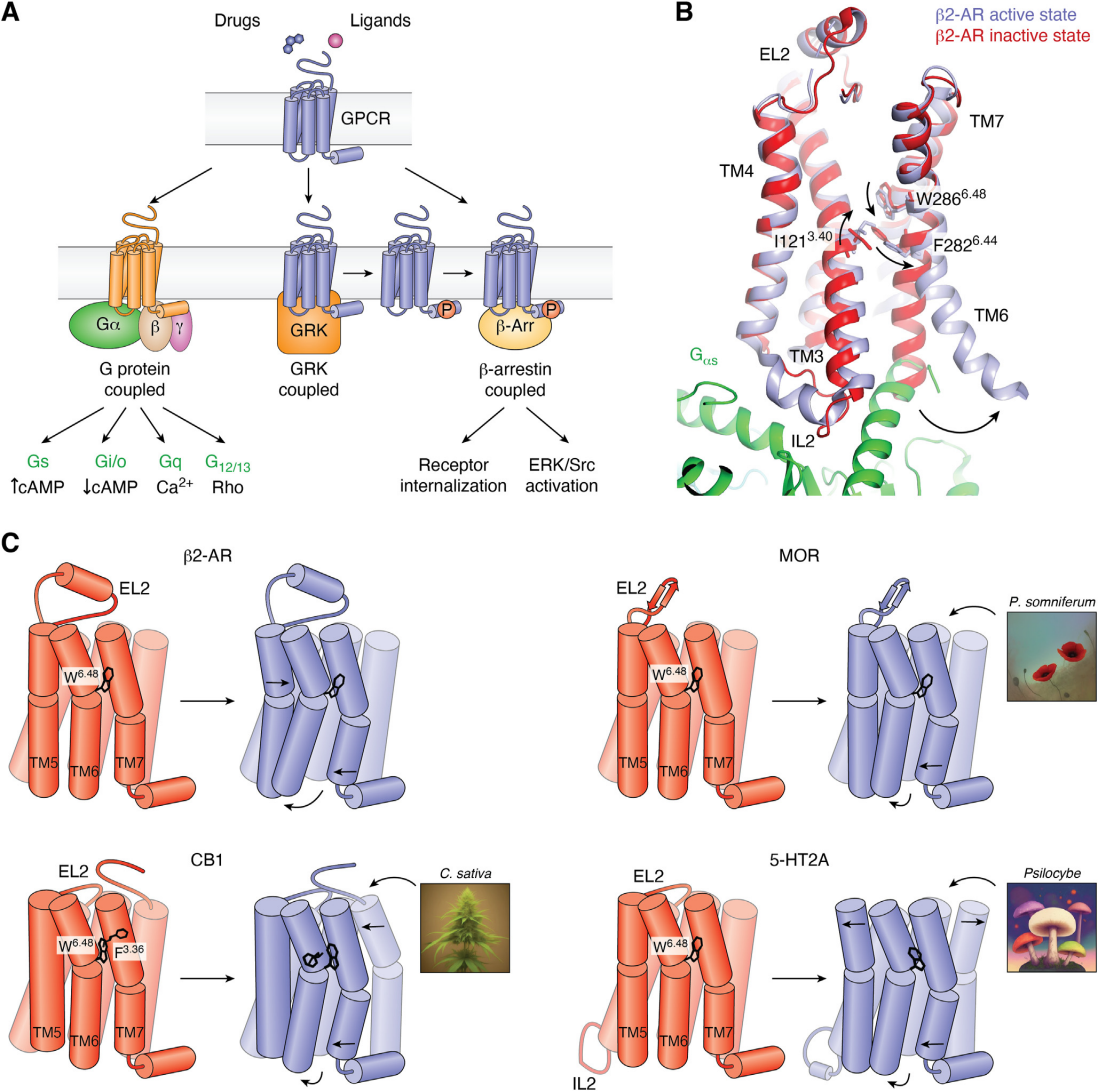
**GPCR activation and signaling.***A*, schematic of GPCR signaling highlighting different transducers including heterotrimeric G proteins (Gα/Gβ/Gγ), GPCR kinases (GRKs), and β-arrestins (β-Arr). Transducer binding and activation modulates secondary messenger (*e.g.*, cAMP, Ca^2+^) levels, activates downstream effectors such as extracellular signal-regulated kinase (ERK), proto-oncogene tyrosine-protein kinase Src (Src), or causes receptor internalization. *B*, superposition of the active (*light blue*, PDB ID: 3SN6) and inactive state (*red*, PDB ID: 2RH1) β2-AR structures reveals activation-related conformational changes largely conserved among class A GPCRs. W^6.48^ located in TM6 connects changes in the ligand-binding site and transducer-binding site. Downward motion of W^6.48^ is connected to coordinated changes of I^3.40^ and F^6.44^ of the P-I-F motif, which links to an outward motion of TM6’s cytoplasmic half. *C*, schematics illustrating differences in the activation mechanisms of MOR, CB1, and 5-HT2A compared to β2-AR according to structural studies. Observed differences, for instance, comprise order-disorder transitions of intracellular loops, changes in the position of TMs, and key residue switches that relate structural changes between ligand- and transducer-binding sites. 5-HT, 5-hydroxytryptamine; β2-AR, β2-adrenergic receptor; CB, cannabinoid receptor; GPCR, G protein–coupled receptor; IL2, intracellular loop 2; MOR, μ-opioid receptor; TM, transmembrane.

## Opioid drugs

### Opioid physiology, pathology, and pharmacology

Opioid receptors mediate both pain-relieving (analgesic) effects, as well as addicting and sometimes lethal effects of opioid drugs, such as morphine and fentanyl. Morphine and derivatives, such as oxycodone and hydrocodone, are widely used as pain medications for conditions such as musculoskeletal back pain (13). The synthetic opioid fentanyl and its derivatives, on the other hand, are routinely used for intubation-related induced comas (14), or to treat cancer-related chronic pain (15).

Opioid medications and their overprescription to patients with chronic pain is believed to have contributed to the start of the opioid epidemic (16). In addition to their analgesic effects, the euphoric effects produced by opioids have facilitated their chronic use. This has led to a buildup of drug tolerance and, ultimately, addiction to the drugs giving rise to opioid use disorder (OUD). This disorder is characterized by cravings that users often try to satisfy with more potent drugs such as fentanyl, which has around a 100-fold increased potency compared to morphine (17). The euphoric effects of opioids are attributed to their modulation of dopaminergic activity in the mesolimbic system, circuitry that has also been associated with withdrawal syndromes produced after cessation of chronic opioid use (18). The most problematic effect of opioids, however, is the disruption of respiration, a lethal side effect that is responsible for the majority of opioid-related deaths (19). Naloxone (NARCAN), a μ-opioid receptor (MOR) antagonist, is typically used as treatment for acute opioid overdose but is not as effective in reversing the effects of more potent opioids such as fentanyl (20). The lack of OUD treatment has caused synthetic opioid–associated deaths to reach epidemic proportions, with fentanyl causing most of the increase in overdose deaths according to National Institute of Drug Abuse (https://nida.nih.gov/research-topics/trends-statistics/overdose-death-rates).

In addition to MOR, other opioid GPCRs include δ- and κ-opioid receptors (21). MOR is identified as the primary molecular target for some analgesic effects as well as the addictive and lethal side effects of opioid drugs, though both δ- and κ-opioid receptors are implicated in modulating these effects (22). Opioids typically act as agonists of MOR, stimulating the activation of inhibitory Gi/o proteins that lower cellular cAMP levels and activate G protein–inward rectifying potassium channels, causing neuronal hyperpolarization, which inhibits excitability. Opioids mediate their analgesic effects by two major mechanisms: (i) by inhibiting peripheral pain signals in nerves, such as C-fibers or neuron clusters such as dorsal root ganglion in the spine and (ii) by blocking the perception of pain and its affective components in the CNS through inhibition of GABAergic interneurons in the periaqueductal gray, the primary pain control center in the brain (22, 23, 24). Sustained stimulation of MOR and chronic G protein–mediated depression of cellular cAMP levels can lead to compensatory upregulation of adenylyl cyclase activity. This thus leads to an increased expression of cAMP-response element binding protein and other cellular adaptations related to G protein signaling (25). At the same time, β-arrestin–mediated internalization of MOR leads to desensitization of opioid signals, less cell surface receptors available for activation, which requires higher opioid doses to maintain comparable cellular inhibition thus driving tolerance.

Interestingly, several preclinical studies suggest that β-arrestin also plays a key role in opioid analgesia, respiratory depression, and the addicting effects of opioid drugs (26). However, there is still considerable ambiguity regarding the precise signaling pathways and mechanisms by which opioids elicit differential signaling or biased agonism (27), which has made it challenging to design safer opioids or novel drugs to combat OUD (5).

### Structural studies of opioids bound to MOR

#### Overall architecture

The first structure of MOR revealed the typical seven-transmembrane (7TM) architecture of a class A (rhodopsin family) GPCR (28), including several conserved motifs across the receptor that have been linked to receptor activation and inhibition (29) (Fig. 1). The large superfamily of 7TM receptors or GPCRs is subdivided into classes from A to F according to sequence similarity. Each class has conserved motifs that play a role in their folding architecture and corresponding activation mechanism. For class A, these motifs include the NPxxY and DRY motifs near the cytoplasmic transducer–binding site, and the P-I-F (30) and CWxP motifs near the receptor core that allosterically connect the ligand-binding site to large scale helical rearrangements (31). Interestingly, MOR possesses a large solvent-exposed ligand entry cavity (31), which can accommodate large endogenous peptides (32). In addition, the receptor’s extracellular loop 2 (EL2) forms a characteristic hairpin structure that may play a role in the initial engagement and ultimate binding of larger peptide ligands such as the endogenous β-endorphin (32).

#### Activation-related features

The first active state structure of an agonist-bound GPCR, that of the β2-adrenergic receptor (β2-AR) bound to Gs (33), serves as an excellent framework to compare activation-related features of MOR, cannabinoid receptor 1 (CB1) and serotonin (5-hydroxytryptamine [5-HT]) receptor 5-HT2A discussed herein (Fig. 1, *B* and *C*). The β2-AR–Gs complex structure reveals rearrangements of conserved motifs along the receptor axis that couple agonists binding to G protein binding. The intracellular side of the receptor is where the most conserved activation-related changes occur. R^3.50^ of the DRY motif changes positions to form a hydrogen bond with Y^5.58^ in transmembrane helix 5 (TM5) (superscripts denote Ballesteros–Weinstein numbering (34)). Moreover, Y^7.53^ of the NPxxY motif in TM7 has an inward motion that causes a reorganization of a water-mediated hydrogen-bonding network that extends to the ligand-binding site (35).

A number of antagonist- and agonist-bound structures of MOR in both inactive and active states have provided insight into the molecular architecture of the receptor and uncovered common mechanisms of activation observed in other rhodopsin-family GPCRs (36). The high-resolution crystal structure of MOR bound to a nanobody revealed this extensive network for the first time, and comparison with the β2-AR active state structure showed nearly identical positioning of conserved residues in the G protein–binding site (36). Similar to β2-AR, agonist/antagonist binding to MOR appears to differentially affect the conformational state of the conserved toggle switch W295^6.48^, propagating structural changes in the binding pocket to larger scale rearrangements on the cytoplasmic face of the receptor (36) (Fig. 1, *B* and *C*). Structures show that conformational changes in W295^6.48^ push on F291^6.44^ located one helical turn below, which is part of the P^5.50^-I^3.40^-F^6.44^ motif (30, 36, 37). As observed in other GPCRs, F291^6.44^ rotates out of the helical bundle toward the membrane, which is further enabled by a conformational change in I157^3.40^. As a result, the conformational change of F291^6.44^ creates torque around TM6, which leads to a swivel motion concluding in an outward movement of TM6’s cytoplasmic tip. This movement opens a crevice on the intracellular site that enables the binding and subsequent activation of transducers such as heterotrimeric G proteins. Despite these conformational changes, agonist- and antagonist-related structural differences in the binding pockets were shown to be rather subtle (Fig. 1*C*), and it has been challenging to unambiguously identify activation- or inhibition-specific receptor–drug interactions (36, 38).

#### Drug binding

**Figure 2:**
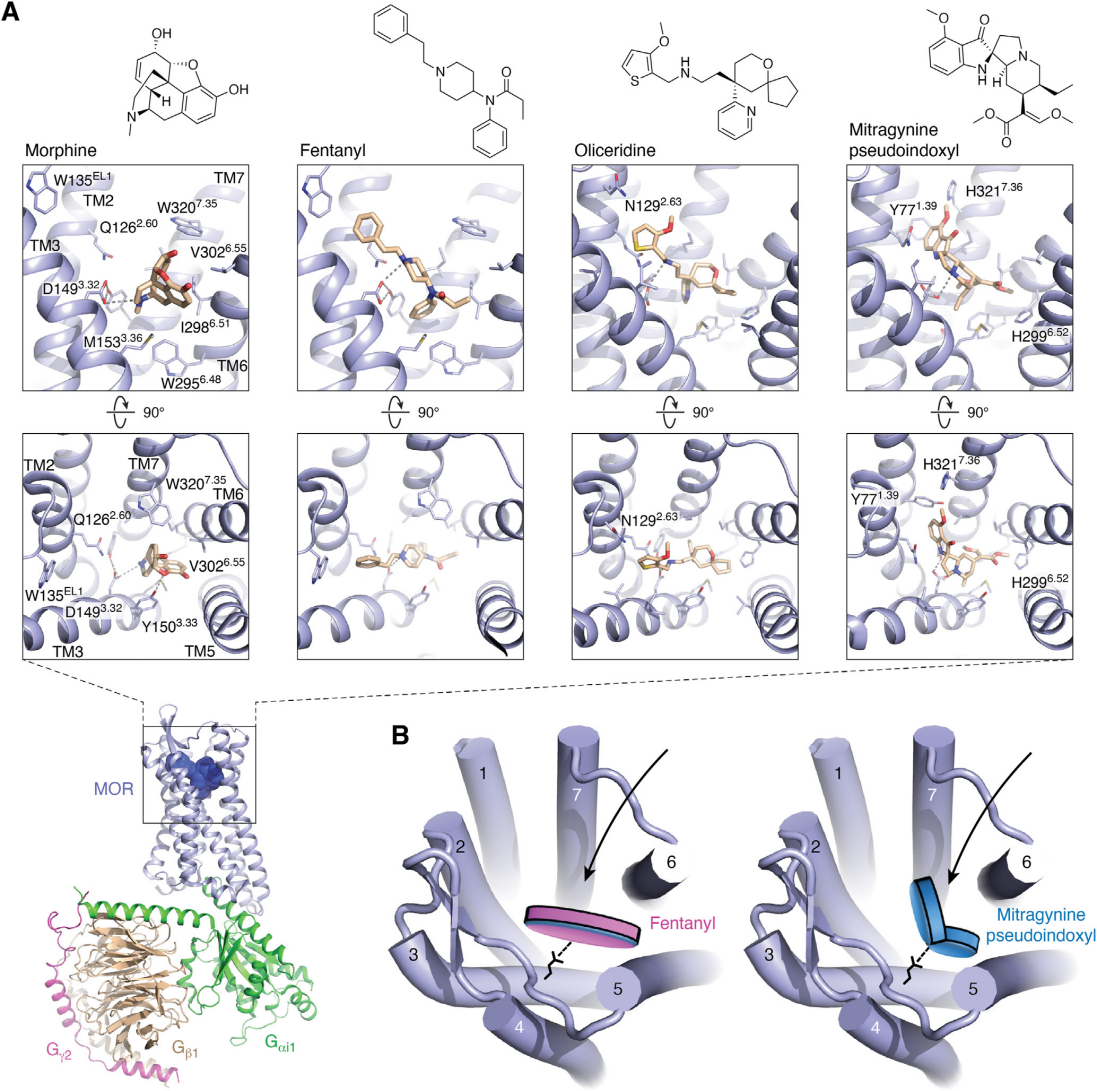
**Structures of opioid drugs bound to the μ-opioid receptor.***A*, overview of the fentanyl-bound MOR-Gi1 signaling complex cryo-EM structure (PDB ID: 8EF5), and chemical structures and close ups of orthosteric-binding pocket bound by morphine (PDB ID: 8EF6), fentanyl (PDB ID: 8EF5), TRV130/oliceridine (PDB ID: 8EFB), and mitragynine pseudoindoxyl (MP) (PDB ID: 7T2G). MOR, Gαi1, Gβ1, and Gγ2 are highlighted in *light blue, green, wheat*, and *magenta*, respectively. *Top*, key side chains and drugs (*light brown*) are shown as *sticks*, and hydrogen bonds and ionic bonds are shows as *gray dashed lines*. *B*, schematic illustrating differences in the binding poses of the opioids fentanyl and MP, the latter of which extends into a distinct pocket near TM7. MOR, μ-opioid receptor; TM, transmembrane.

Structural studies elucidated the precise location and three-dimensional architecture of the orthosteric-binding pocket over ten years ago (29). The publication of a structure of small peptide DAMGO ([D-Ala2, N-MePhe4, Gly-ol]-enkephalin) bound to MOR in complex with the heterotrimeric G protein Gi in 2018, and the more recent structures of opioid receptors bound to endogenous peptides, have provided further insight into mechanisms of MOR-specific peptide-mediated signaling (32, 39). However, the precise binding modes and receptor interactions of the small molecule MOR agonists fentanyl and morphine were only uncovered recently (5, 40). Fentanyl and morphine are chemically different and bind to MOR in structurally distinct modes (Fig. 2*A*). Both drugs form a conserved ionic interaction with D149^3.32^, but morphine is primarily wedged between residues of TM3, TM5, TM6, and TM7, whereas fentanyl appears to stretch across the entire binding pocket forming additional interactions with TM2. Morphine’s binding pose is further stabilized by a hydrogen bond with Y150^3.33^, and the drug is located directly above W295^6.48^. Fentanyl, on the other hand, appears to form direct π–π interactions with its phenyl group wedged between residues W295^6.48^ and Y328^7.43^, whereas morphine’s methyl substituent only forms minor hydrophobic contacts in this pocket (Fig. 2*A*). Fentanyl additionally possesses a benzyl group that extends toward a crevice between TM2 and TM3, where it forms a face-to-edge contact with tryptophan W135^EL1^ in EL1 that appears critical for fentanyl’s superior affinity and potency (as determined by EC50) (5). Together, these additional contacts explain how fentanyl is 50- to 100-fold more potent than morphine, which directly relates to fentanyl’s disproportionally high lethality (41). Structure-based studies further shed light on the increased potency of additional fentanyl derivatives such as lofentanil or carfentanil, the latter of which is used in veterinary medicine to sedate large animals such as elephants. Most fentanyl derivatives contain modified piperidine moieties, and both structural studies of lofentanil-bound MOR as well as computational docking and simulations suggest that these substituents do not change the overall binding pose of the fentanyl scaffold (5, 40). Instead, the piperidine substituent forms additional contacts with I298^6.51^, W320^7.35^, and I324^7.39^, in TM6 and TM7, which appear responsible for their increased potencies compared to fentanyl (5).

### Implications for drug pharmacology and development of therapies

Together, these structural insights not only greatly advance our understanding of general opioid receptor function but also directly highlight how morphine and fentanyl bind and activate MOR. As MOR and other opioid receptors directly mediate much of the physiological effects of morphine, fentanyl, and other opioid drugs, they are the principal targets of drug development to mitigate OUD and other side effects associated with these drugs. For instance, NARCAN, which competes for binding of the receptor, is a primary tool to rapidly reverse the effects of an acute opioid overdose. By contrast, the synthetic MOR partial agonist methadone and the morphinan partial agonist buprenorphine (formulated in combination with naloxone as Suboxone) are used to manage cravings and withdrawal symptoms. However, methadone and buprenorphine both have milder, yet similar side effects and abuse potential as fentanyl and other opioids (42). This underscores the need for novel, more efficacious medications for the treatment of opioid addiction and management of withdrawal symptoms (43). Recently, significant effort has been dedicated to designing novel opioid -based analgesics with reduced side effects (44, 45). Several studies and drug development campaigns have focused on developing novel MOR drugs that target distinct signaling pathways. Previously, mouse KO studies indicated that analgesic effects are mostly associated with G protein signaling, whereas β-arrestin–mediated signaling could be responsible for the development of tolerance, addiction, as well as the lethal side effects of opioid drugs (46, 47, 48). Much effort has thus been dedicated toward developing G protein–biased ligands devoid of β-arrestin activity (48). A computational ligand discovery campaign produced one such compound, PZM21, by leveraging MOR structural data. PZM21 not only shows weaker MOR β-arrestin recruitment *in vitro* but also displays improved safety profiles in preclinical animal studies that probe respiratory depression (49). Similarly, SR-17018 and TRV130 are also MOR selective G protein–biased ligands with significantly reduced β-arrestin recruitment activity that exhibit reduced dependence and respiratory depression in rodent models (48, 50). Recent structural studies of PZM21, SR-17018, and TRV130 show reduced interactions with MOR residues in TM6 and TM7 compared to morphine and fentanyl (Fig. 2*A*), which activate both G protein and β-arrestin–mediated signaling (5). It was thus suggested that interactions with TM6 and TM7 residues are critical for stabilizing MOR conformations that potently engage β-arrestin, which mirrors similar findings at other GPCRs (30). After initial rejection by the Food and Drug Administration, TRV130 has recently made it to market under the name Oliceridine, though it should be noted that the drug still exhibits considerable respiratory side effects in clinical studies and is only indicated for managing moderate to severe acute pain (51, 52). Follow-up studies have since indicated that elimination of β-arrestin–mediated events does not necessarily lower opioid side effects and suggest that G protein signaling plays a major role in adverse events (27, 53, 54). In light of these findings, it was suggested that low intrinsic efficacy rather than G protein bias is responsible for increased therapeutic windows of novel opioid compounds (55, 56).

In the absence of novel opioid drugs with improved safety profiles, patients have turned to the use of Kratom extract from the tree *Mitragyna speciose* to manage chronic pain. Although the safety of this extract has been controversial and several deaths associated with its use have been reported (57), it does show analgesic effects and alleviates opioid withdrawal symptoms (58). Mitragynine and several analogs are the key compounds in Kratom that feature MOR agonism and are at the center of current drug discovery efforts due to their potentially attenuated side effects (40, 59, 60). Interestingly, mitragynines have recently also been described as G protein–biased compounds (61), though other studies attribute their increased safety profiles to partial agonism (60). It should be noted, however, that mitragynines show considerable affinity at other receptors such as serotonin and adrenergic receptors, which likely play a role in the physiological effects of these compounds (62).

Recently, structural studies of the analog mitragynine pseudoindoxyl (MP) revealed its binding mode at MOR, uncovering a binding pose distinct from that of morphine, fentanyl, and other opioid ligands, including the G protein–biased drug oliceridine (Fig. 2*A*) (40). While MP forms the conserved ionic bond with D149^3.32^, its pseudoindoxyl group extends toward a crevice formed by TM1, TM2, and TM7, forming largely hydrophobic interactions. This binding mode is unique to MP and was not observed for morphine, fentanyl, oliceridine, or any of the other experimental opioid drugs probed in structural studies (Fig. 2, *A* and *B*). This binding mode was suggested to potentially explain MP’s distinct pharmacological activity, such as lower efficacy at MOR compared to morphine and fentanyl (40) (Fig. 2*B*).

Despite the socioeconomic burden of the opioid epidemic and the devastating side effects of current opioids, these drugs currently still remain the most effective analgesics. The studies reviewed here provide a glimpse into the current landscape of developing safer analgesics and novel medications for the treatment of OUD. Moreover, atomic level insights from structures combined with molecular inquiries into their pharmacological mechanisms have already led to novel tool compounds that promise to facilitate drug development efforts.

## Cannabinoid drugs

### Cannabinoid physiology, pathology, and pharmacology

The lipid-activated cannabinoid receptors CB1 and CB2 mediate most of the physiological effects of compounds found in the plant *Cannabis sativa* (63), a widely used drug of abuse with therapeutic potential for a variety of ailments. Endogenously, CB1 and CB2 are activated by modified lipids termed endocannabinoids, most notably anandamide and 2-arachidonoylglycerol (64). The endocannabinoid system not only plays a role in the regulation of cognitive processes, appetite, and mood, which are mostly mediated by central CB1 receptors, but also in various activities of the immune system, by means of CB2 activation (64).

Cannabis or marijuana is a psychoactive drug that can create a complex perceptual sensation colloquially referred to as being “high” or “stoned,” which includes a range of pleasurable and dysphoric effects in users. In addition to recreational use, cannabis has several therapeutic applications, ranging from anxiety to pain and even immunological disorders (65, 66). Antiemetic and analgesic properties are of particular interest for the treatment of nausea during chemotherapy or for chronic pain, respectively (67). These therapeutic effects have significantly contributed to recent measures to decriminalize or even legalize the drug in several countries, including the United States.

Like opioid receptors, CB1 and CB2 signal through the Gi/o family of G proteins (66). In the CNS, CB1 is mostly expressed in presynaptic terminals and its activation thus reduces neuronal excitability and suppresses neurotransmission. Not surprisingly, the analgesic effects of cannabis have been in part ascribed to inhibition of pain signals at the spinal and supraspinal level such as at the dorsal horn and the periaqueductal gray (68). Despite its therapeutic effects, the psychoactive effects of cannabis typically result in severely altered or impaired perception and coordination, and, in some extreme cases, even hallucinations (69). These properties raise significant safety concerns regarding its use as a medication and as a recreational drug. On the other hand, studies show that the consumption of cannabis is not more harmful than that of any other legal recreational drug such as alcohol or nicotine (70). However, marijuana consumption has considerable addiction liability, with studies estimating 10 to 30% of users becoming addicted to these drugs (71, 72). Cannabis use disorder (CUD) is poised to have a severe societal impact, given its place as the third most frequently used recreational drug after alcohol and tobacco as of 2021, and increasing momentum in legalization of its use and sale throughout the United States (73). The appearance of extremely potent synthetic cannabinoids, such as FUBINACAs and PINACAs, add concern due to the almost comatose state they produce in users (74). These so-called “zombie drugs” pose a severe public health risk and highlight the challenge of generating safe CB1-targeted medications (74).

Similar to opium, cannabis contains over 100 different cannabinoid compounds (75). The most studied cannabinoids are Δ9-THC and its isomer Δ8-THC, which is slightly weaker at CB1. These compounds are generally responsible for the psychoactive effects associated with cannabis use. THC shows partial agonist activity, both *in vitro* (76) and *in vivo* (77), while synthetic cannabinoids such as FUBINACAs are full agonists with considerably higher potency than THC (78). Administration of the CB1 inverse agonist rimonabant–an antiobesity drug withdrawn from sale due to severe psychiatric side effects (79)–largely blocks the intoxicating psychoactive effects of THC (80). Another major component of cannabis is cannabidiol (CBD), a nonintoxicating compound with recent Food and Drug Administration approval for the treatment of epilepsy and other seizure disorders (Epidiolex) (81). While CBD has also been associated with a variety of other health benefits, it should be noted that many of these claims remain under investigation. CBD has been alternately reported as either an antagonist, inverse agonist, or negative allosteric modulator at both CB1 and CB2 receptors (82), though CBD seems to have low-micromolar affinity for both (83). Additionally, CBD appears to be an inverse agonist of the orphan receptors GPR3 and GPR6, which is not observed for THC (84). CBD also acts *via* more diverse effectors such as transient receptor potential cation channels (*e.g.*, TRPV1) and paradoxically appears to block release of proinflammatory factors *via* CB2 stimulation (68).

More broadly, a number of orphan GPCRs including GPR3, GPR6, GPR12, GPR18, and GPR55 have been putatively linked to cannabinoid, and particularly CBD, pharmacology (85), although conflicting reports exist regarding compound potency and efficacy at these lesser studied targets. Most notable amongst these is GPR55, which has been proposed as a potential third cannabinoid receptor due to its activation by THC and the endogenous cannabinoid receptor agonist anandamide (86, 87). However, this receptor also potently responds to lysophosphatidylinositol, and thus its status as a dedicated cannabinoid receptor has been disputed. It is thus quite likely that with additional work, the current model of cannabinoid action and physiology will have to be revised substantially. In the interim, the current literature suggests that the higher affinity and efficacy of synthetic cannabinoids (and their metabolites) at CB1 could contribute to their severe toxicity and overdose potential (88), which may also conversely explain the lack of psychoactive effects associated with CBD. However, alternative mechanisms could explain the unique pharmacological activity of the different compounds. For instance, THC appears to show significantly reduced efficacy in β-arrestin–mediated events such as receptor internalization when compared to AMB-FUBINACA or the prototypical CB1 agonist probes CP55,940 and WIN55,212-2 (78). This pharmacological ambiguity in the mechanisms of action of synthetic and plant cannabinoids requires further study at the level of receptor–ligand interactions. Molecular insights from these may best address how to therapeutically separate adverse toxicity from desired antinociceptive and anxiolytic effects.

### Structural studies of CB1 bound to cannabinoid drugs

Over the past years, several crystal and cryoEM structures of CB1 (and CB2) in inactive, intermediate-active, and active states coupled to G proteins have illuminated key features of drug binding and receptor activation. As CB1 is the principal target of THC’s psychoactive effects, we summarize the findings from the structural work related to this receptor.

#### Overall architecture

**Figure 3:**
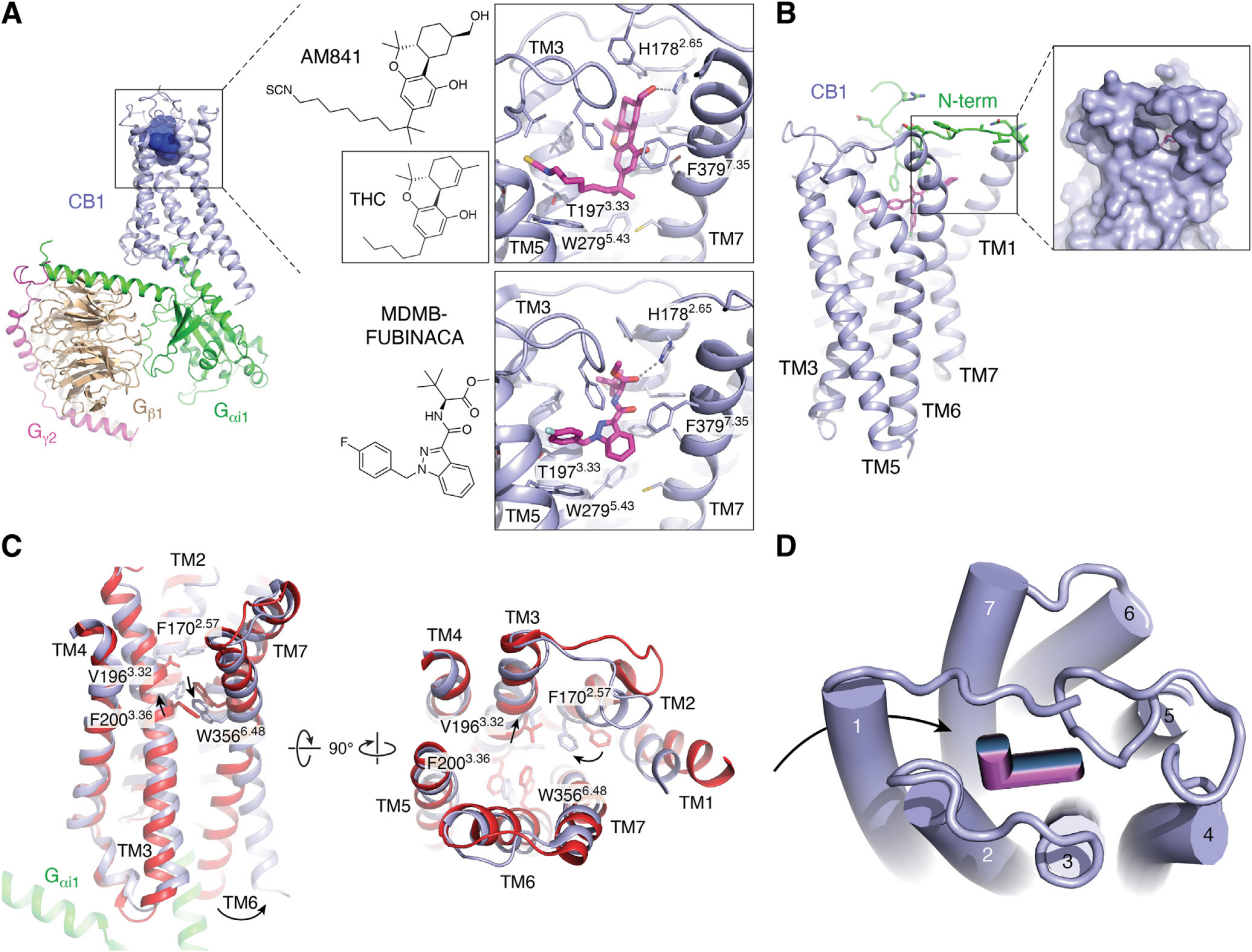
**Structural insights into the molecular actions of cannabinoid drugs.***A*, overview of G protein–bound CB1-agonist complex (PDB ID: 6KPG) with the receptor, Gαi1, Gβ1, and Gγ2 highlighted in *light blue, green, wheat*, and *magenta*, respectively. Chemical structures and close ups of cannabinoid drugs AM841 (PDB ID: 6KPG) and MDMB-FUBINACA (PDB ID: 6N4B) bound to the CB1 orthosteric pocket, and inset shows chemical structure of THC by comparison. Drugs (*magenta*) and side chains are shown as *sticks*, and hydrogen bonds and ionic bonds are indicated by *gray dashed lines*. *B*, membrane view of CB1 showing 7TM architecture (*light blue*) (PDB ID: 5TGZ). Residues of the N terminus are shown in *green* and bound drug AM6538 is shown in *magenta*. Zoom-in shows gap in TM1-TM7 interface, which likely serves as the entry pore for hydrophobic CB1 ligands from within the membrane. *C*, proposed activation of CB1 elucidated by the overlay of inactive state (*red*, PDB ID: 5TGZ) and G protein–bound (*green*) active state (*light blue*, PDB ID: 6KPG) involves inward motion of aromatic residues in TM2, followed by the pairwise motion of F200^3.36^ and W356^6.48^, designated as the twin-toggle switch. *D*, schematic illustrates the L-shape binding mode of cannabinoid drugs, and the reported receptor entry of cannabinoid ligands from the membrane *via* an opening of the 7TM bundle. 7TM, seven-transmembrane; CB, cannabinoid receptor; THC, tetrahydrocannabinol.

CB1 is a class A GPCR, featuring a typical 7TM architecture and the same conserved signaling motifs discussed prior. However, while many GPCRs such as MOR display a solvent-exposed entry cavity needed for effective binding of soluble opioid peptides and small molecules, CB1 features an extensive extracellular surface shielding its hydrophobic ligands from solvent exposure and extending into the binding pocket (Fig. 3, *A* and *B*) (89, 90, 91). As observed for other lipid-activated GPCRs, the receptor’s N terminus folds over the 7TM bundle to enclose the ligand pocket. In the structures of CB1 bound to the antagonists taranabant and AM6538 (a rimonabant analog), the N terminus reaches into the 7TM core to directly contact the ligands. Hydrophobic cannabinoids are thus proposed to enter the receptor within the membrane through an opening between TM1 and TM7 (Fig. 3*B*) (90). Moreover, the structures further reinforce how chemically diverse ligands interact mostly with phenylalanine residues in EL2, TM7, the N terminus, and other hydrophobic residues lining the presumed orthosteric and nearby pockets (Fig. 3*A*) (91).

#### Activation-related features

Available structures of CB1 in both active and inactive states provided new insights into both CB1-specific and general features of receptor activation shared by other rhodopsin-family GPCRs (Fig. 3*C*) (92, 93, 94). For instance, agonist binding, as observed in other receptors, causes a contraction of the binding site located near the extracellular side of the 7TM bundle, with studies reporting as much as a 53% decrease in binding pocket volume (95). The conformational changes responsible for this contraction seem rather unique to CB1, as inward movements of the extracellular tips of TM1 and TM2 appear in large part responsible for the decrease in pocket volume. Structures of CB1 bound to taranabant or AM6538 show that antagonists interact with TM1 residues, likely preventing inward movements and subsequent receptor activation (89, 90). Strikingly, when compared to taranabant- or AM6538-bound CB1, the receptor’s N terminus does not enter the ligand-binding pocket in the agonist bound states (92, 95). Instead, it forms interactions with other loops in the extracellular surface but occasionally still directly interacts with the ligand such as a hydrophobic interaction between F108^Nterm^ and the THC analog AM841 (92).

Activation of CB1, similar to other class A GPCRs, is characterized by large-scale helical changes in TM6 and TM7 as described previously, although several conformational changes appear to be specific to CB1. For instance, comparison of active- and inactive-state CB1 structures suggests that agonist binding and related inward movements of TM1 and TM2 lead to a rearrangement of key residues in the binding pocket that propagate conformational changes between orthosteric pocket and transducer-binding site. Specifically, the inward movements of TM1 and TM2 cause a rotation in TM2 that moves F170^2.57^ toward the center of the binding pocket, where it pushes on V196^3.32^ in TM3. As a result, TM3 shifts toward the extracellular space by nearly 2 Å, which appears to cause a rotamer switch in F200^3.36^ with the sidechain relocating from a crevice between TM5 and TM6 toward TM1, TM2, and TM7. This change allows the conserved toggle switch W356^6.48^ to move toward TM5, leading to the characteristic outward motion of the helix at the cytoplasmic site. The pairwise motion of F200^3.36^ and W356^6.48^ characteristic of cannabinoid receptors has led to their designation as a twin-toggle switch (95, 96) (Figs. 1*C* and 3C).

#### Drug binding

The structures of agonist-bound CB1 further provide detailed insight into the binding mode of novel cannabinoids such as MDMB-FUBINACA, or the THC-analog AM841, and thereby provide insights into the putative binding mode of THC (Fig. 3*A*). Overall, all agonists assume an L-shape in the binding pocket with the two perpendicular “segments,” laying parallel and orthogonal to the membrane plane (Fig. 3, *A* and *D*). Of note, CB1 agonists do not seem to interact directly with TM1 compared to antagonists. The alkyl chain of AM841 and the fluorophenyl substituent of MDMB-FUBINACA are located at the bottom of the binding pocket and extend toward the interface of TM3, TM4, and TM5. The substituted indazole moiety of MDMB-FUBINACA and the tricyclic THC moiety of AM841 pack against TM2 and TM7 residues, with additional interactions with EL2 that covers the binding pocket from the solvent site. In accordance with the hydrophobic nature of these drugs and their likely passage into the binding pocket *via* the membrane plane, most observed drug–receptor interactions are hydrophobic and involve phenylalanine residues in CB1’s ligand-binding surface. One notable exception is the presence of a hydrogen bond between H178^2.65^ and both AM841 and MDMB-FUBINACA (Fig. 3*A*).

### Implications for drug pharmacology and development of therapies

Structural insights into cannabinoid receptors bound to both research chemicals and drugs of abuse have greatly expanded our understanding of the molecular underpinnings of cannabinoid pharmacology. For instance, these studies provide a structural framework for how modifications of THC’s alkyl chain length and composition modulates drug potency and, to some extent, signaling efficacy (97). The structures suggest that the alkyl chain is tightly threaded through a narrow and hydrophobic channel, explaining how longer chains up to eight carbons correlate with an increased affinity and potency, before further extension becomes detrimental. Similar inferences can be made about the reported effects of ω-substitutions such as AM841’s isothiocyanate group, addition of branching, or rigidification of the chain (97). Furthermore, the structure of the CB1-MDMB-FUBINACA complex has provided a structural context for structure-activity relationship data of several synthetic cannabinoid full agonists. For instance, the structures now reveal how differential interactions with TM3, TM5, and EL2 residues likely explain the different potencies of indazole-based PINACA, CHMICA, and FUBINACA compounds (98). The structures of CB1 bound to MDMB-FUBINACA and AM841 show compound interactions with F200^3.36^, which was identified as a key switch linked to receptor activation (92, 93). MDMB-FUBINACA and other indazole designer drugs form an aromatic stacking interaction with F200^3.36^, highlighting the role of this residue in the efficacy and toxicity of synthetic cannabinoid full agonists (93). Since THC lacks the 1′,1′-gem-dimethylheptyl group of AM841, the structural evidence suggests that direct interactions with F200^3.36^ are responsible for the increased efficacy of AM841 and potentially explain THC’s partial agonist behavior.

Taken together, structural studies of CB1 have thus provided a foundation from which to further dissect cannabinoid pharmacology in molecular detail. These are timely studies considering ongoing legalization efforts, as well as the risks posed by consumption of cannabis and particularly synthetic cannabinoids. Beyond acute impairments following drug consumption, CUD and associated long-term detrimental effects on mental health have become a severe public health liability, and require effective treatment options. The use of CB1 antagonists such as rimonabant generally produces anxiety, depression, and even suicidality (99), hindering their potential use in treating CUD. It has been suggested that this may be due to rimonabant’s inverse agonism, and that neutral antagonists may provide therapeutic effect without the associated adverse events (79). Interestingly, recent clinical trials found that 400+ mg of CBD appear to significantly lower cannabis use in patients with CUD (100), but further studies are required to better understand the effects of this drug. Structural and molecular studies will undoubtedly also facilitate the generation of novel probes and drug candidates with improved safety profiles. In fact, additional structural studies have elucidated binding sites and mechanisms of both positive and negative allosteric modulators of CB1 (94, 101), which are generally regarded as safer alternatives to orthosteric drugs (102).

## Psychedelic drugs

### Psychedelic physiology, pathology, and pharmacology

Psychedelics such as the synthetic drug LSD are known to exhibit significant mind-altering effects and a substantial impact on consciousness. This class of drugs also includes the natural substances psilocybin (found in *Psilocybe Cubensis*, colloquially “magic mushrooms”), dimethyltryptamine (found in Ayahuasca along with monoamine oxidase inhibitors), and mescaline (found in the *Lophophora williamsii* peyote cactus), which are used in shamanic rituals among several indigenous populations (7). The acute effects of psychedelics include powerful impairments of perception such as audio-visual distortions and synesthesia, often collectively referred to as hallucinations. Historically, these drugs have also been referred to as psychotomimetics, which stems from the original comparison of aspects of psychedelic-induced altered state of consciousness to psychosis-like states. After a dearth of psychedelic research following their classification as scheduled substances in the United States in 1970 (7, 103), interest in these compounds has recently resurged due to their therapeutic potential for the treatment of depression, anxiety, substance-use disorders, and other neuropsychiatric maladies (104, 105, 106).

Regarding safety, little evidence exists to suggest that classic psychedelics such as LSD or psilocybin cause acute physical toxicity or harm to the user, although they do impair the user’s perception. Perceptual dysfunction, for example, judgment of speed and distance when operating a motor vehicle, can cause severe harm to the user or others (7), but reports of psychedelics causing permanent psychosis or episodes of recurrent psychotic-like mental states (colloquially “flashbacks”) have not been conclusively substantiated (7). In recent years, however, the incidence of persistent perceptual aberrations has increased in a subset of hallucinogen users. These symptoms are collectively termed hallucinogen-persisting perception disorder. This disorder is generally very rare and challenging to diagnose but has received more attention in recent years with the increasing popularity of psychedelics (107, 108, 109). It should also be noted that severe toxicity, including fatalities, has been reported for synthetic designer psychedelics such as Bromo-dragonfly (a drug related to the phenethylamine class) (110).

Compared to other substances such as opioids, psychedelics typically do not activate the mesolimbic system, and are thus not reported to be addictive in the traditional sense (*i.e.*, they do not cause compulsive drug seeking). In fact, psychedelic experiences (colloquially “trips”) are often reported as intense and not always comfortable, and users frequently abstain from seeking another trip for a prolonged time. However, selfadministration of LSD has been observed in nonhuman primates, albeit at low frequency compared to classical drugs of abuse (111). While still being classified as a drug of abuse, the divergent behavioral and pharmacological effects of psychedelics reduce their abuse potential and consequently pose much less of a danger to public health (70) compared to other drugs such as opioids.

Several lines of research have identified the serotonin receptor 5-HT2A as the key mediator of the perceptual effects of psychedelics (7). However, other 5-HT receptors such as 5-HT1A and 5-HT2C are involved in modulating the complex psychological and physiological effects of psychedelics (112, 113). Generally, studies point to the cortex as the main site of action for acute psychedelic-like effects (7, 114). For instance, the prefrontal cortex, where 5-HT2A receptors are highly expressed, serves as a hub for processing sensory information and controlling higher executive function *via* layer V pyramidal neurons. At the cellular scale, most psychedelics act as partial agonists at 5-HT2A receptors and appear to stimulate Gq-mediated signaling pathways. Moreover, both *in vitro* and *in vivo* studies suggest that 5-HT2A coupling to β-arrestins plays a key role in the behavioral effects of psychedelics (115, 116, 117). This includes recent findings using psychedelic-derived compounds (118, 119, 120). However, there is currently no consensus as to which 5-HT2A signaling pathways are critical for psychedelic effects. Addressing this question is further complicated by the complexity of the psychedelic experience, which includes alterations of perception, emotional state, cognition, and volition that cannot be easily recapitulated or meaningfully tested in animal models (121). Conceivably, these alterations can arise from the different brain regions involved in these processes, followed by activation of different receptors and signaling pathways psychedelics are known to modulate (122). Further complications exist in that different but structurally similar psychedelics can cause different sets of subjective effects, which further differ depending on the mindset of the user and the affective valence of the environment the user inhabits (“set and setting”). To understand the complex effects of psychedelics at the molecular scale, careful examination of how they interact with their cognate target receptor(s) and engage distinct signaling pathways is required.

### Structural studies of psychedelics bound to serotonin receptors

Over the past 10 years, structures have become available for all human 5-HT receptors bound to a wide variety of drugs (30, 117, 123, 124, 125, 126, 127, 128, 129). These studies have not only shed light on the general architecture and activation mechanisms of G protein–coupled 5-HT receptors but also uncovered molecular features of how psychedelics bind to and elicit their unique pharmacological effects. Here, we focus on 5-HT2A, 5-HT2B, and 5-HT2C because multiple structures of these receptors in complex with a range of transducers and ligands are available.

#### Overall architecture

**Figure 4:**
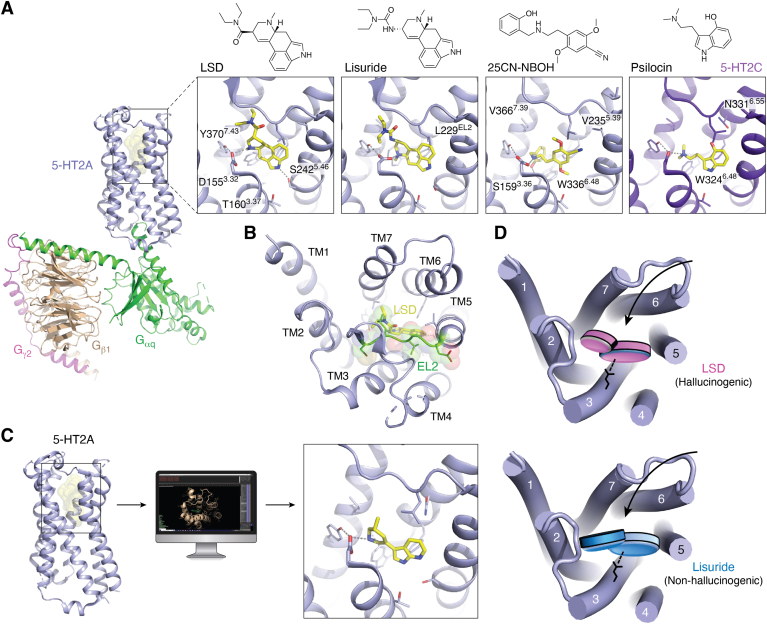
**Structural studies of psychedelics and development of novel 5-HT2A agonists.***A*, overview of the 25CN-NBOH–bound 5-HT2A–Gq signaling complex cryoEM structure (PDB ID: 6WHA), with the receptor, Gαq, Gβ1, and Gγ2 highlighted in *light blue, green, wheat,* and *magenta*, respectively. Close ups of 5-HT2A (*light blue*) and 5-HT2C (*purple*) orthosteric-binding sites showing binding poses of LSD (PDB ID: 6WGT), lisuride (PDB ID: 7WC7), 25CN-NBOH (PDB ID: 6WHA), and psilocin (PDB ID: 8DPG). Side chains and drugs (*yellow*) are shown as *sticks*, and *gray dashes* indicate hydrogen bonds and ionic interactions. *B*, extracellular view of the LSD-bound 5-HT2A orthosteric site reveals extracellular lid (*green*) formed by EL2 that covers the binding site. *C*, computational structure–guided ligand discovery generates a novel 5-HT2A agonist, (R)-69, whose binding pose was experimentally determined (PDB ID: 7RAN). *D*, schematic illustrates the distinct binding poses of the chemically related compounds LSD and lisuride that have been proposed to play a role in the distinct pharmacological effects of the drugs. 5-HT, 5-hydroxytryptamine; EL, extracellular loop; LSD, lysergic acid diethylamide.

Structures of the 5-HT2A receptor in inactive and active states provide a general overview of the architecture of the receptor (117, 118, 120, 130) (Fig. 4*A*). As with other aminergic GPCRs, the orthosteric-binding pocket of the receptor is partially shielded from the extracellular space by EL2, which connects TM4 and TM5 with TM3 *via* a disulfide bond between C^45.50^ and C^3.25^ and stretches outside the TM bundle (30, 131, 132) (Fig. 4, *A* and *B*). EL2 acts as a lid that may be responsible for LSD’s slow association and dissociation kinetics observed in 5-HT2A and 5-HT2B (116). Another noticeable feature of 5-HT2A is its intracellular loop 2, which adopts a helical secondary structure in the G protein–coupled state, in contrast to the inactive state (117) (Fig. 1*C*). Conformational differences in the positioning of this helix have been suggested to distinguish hallucinogenic from nonhallucinogenic 5-HT2A agonists (133). The orthosteric-binding pocket is built around the aspartate residue D^3.32^ in TM3, which is conserved in aminergic and opioid receptors (see above). In aminergic receptors, D^3.32^ forms the anchoring salt bridge with the basic amine group found in monoamine neurotransmitters. For 5-HT2A, this salt bridge is necessary to bind psychedelics and other ligands, including the antagonists and atypical antipsychotics risperidone and zotepine (130). The pocket is further comprised of residues in TM5, TM6, and TM7, including the conserved phenylalanines F^6.51^ and F^6.52^ in TM6 that stabilize aromatic scaffolds, such as 5-HT’s indole, *via* π–π interactions (131).

#### Activation-related features

Superposition of the inactive- and active-state structures of 5-HT2A uncovers conserved features of receptor activation. Unlike other rhodopsin-family GPCRs such as CB1, agonist-mediated 5-HT2A activation does not appear to be initiated by a contraction of the orthosteric-binding site. In fact, the comparison of multiple available 5-HT2A structures bound to chemically diverse compounds with different pharmacological properties only shows minor changes in the binding pocket. Though it should be noted that a structure of a drug-bound 5-HT2A–Gq complex displays a modest, possibly drug-specific, expansion of the ligand-binding pocket (Fig. 1*C*) (117). The detailed structural features of the ligand or ligand-mediated interactions that can differentiate 5-HT2A agonists from antagonists thus remain largely unknown (117).

Overall, 5-HT2A activation features similar conformational changes as those described for β2-AR above. That includes conformational changes around the toggle switch W336^6.48^, which sits directly “below” the orthosteric ligand–binding pocket (117), and is pushed by at least 2 Å toward the receptor core depending on the ligand.

#### Drug binding

Although the conformational differences in the orthosteric pockets of active- and inactive-state 5-HT2A appear subtle, the publication of a plethora of drug-bound receptor structures has illuminated key features of how psychedelics bind to serotonin receptors. The first structural insights came from a structure of the 5-HT2B receptor bound to LSD (116). LSD belongs to the class of ergolines, rigidified tryptamines that are chemically closely related to serotonin. The structure of LSD-bound 5-HT2B elucidated the binding pose of the ergoline scaffold within the orthosteric-binding pocket. The indole core of the drug is wedged between hydrophobic side chains of TM3 and TM6 and extends toward TM5, a key area implicated in the activation of many aminergic GPCRs (33, 37). LSD’s diethyl moiety interacts with TM3 and TM7 in an extended binding pocket, which contributes to potency and efficacy bias between transducers. The importance of this interaction for LSD’s pharmacology was further supported using stereoselective LSD analogs that demonstrated efficacy differences in Gq activation at 5-HT2B and potency differences in β-arrestin recruitment at both 5-HT2A and 5-HT2B (116, 117). The structure of LSD bound to 5-HT2A shows a similar drug-binding pose to that at 5-HT2B. The compound adopts a near-identical conformation with the key difference that its indole nitrogen forms a hydrogen bond with S242^5.46^ in TM5, which appears to contribute to LSD’s slow dissociation kinetics at 5-HT2A (117) (Fig. 4*A*). Recent cryo-EM structures of transducer-free 5-HT2B, or 5-HT2B in complex with mini-Gq or β-arrestin1, show that LSD adopts a similar binding pose in all three structures (134).

In 2020 the first cryo-EM structure of a 5-HT2A signaling complex became available and elucidated the unique binding pose of synthetic psychedelic 25CN-NBOH, a compound of the phenethylamine/N-benzyl class (117) (Fig. 4*A*). 25CN-NBOH forms the conserved salt bridge with D155^3.32^, and its phenethylamine scaffold is located in the orthosteric-binding pocket overlapping with LSD’s ergoline scaffold. Aside from the absence of any notable hydrogen bonds in 5-HT2A’s orthosteric pocket, the most intriguing finding is that the drug’s hydroxybenzyl moiety is accommodated in an extended pocket adjacent to the orthosteric site located closer to the receptor’s TM core. Strikingly, while the hydroxyl group forms a hydrogen bond with S159^3.36^, the benzyl group displaces the side chain of the toggle switch W336^6.48^, which could explain the drug’s high efficacy (30, 37).

More recently, structures of 5-HT2A and 5-HT2C in complex with psilocin, the active metabolite of psilocybin, have become available (118, 135). Since psilocin’s unusual binding pose at 5-HT2A is likely due to receptor modifications, we herein only review the 5-HT2C–Gq complex cryo-EM structure, which likely reflects the active state psilocin binding pose (Fig. 4*A*). Comparison of structures confirms that the tryptamine psilocin binds to the same orthosteric pocket in 5-HT2C that accommodates LSD and 25CN-NBOH in 5-HT2A. Other than a conserved ionic bond with D134^3.32^, psilocin does not appear to form any hydrogen bonds between its characteristic 4-OH group and the receptor. Instead, the drug seems to be stabilized by similar π–π stacking interactions between its indole scaffold and phenylalanine residues in TM6, as observed for other aromatic 5-HT receptor ligands (131).

Together, the elucidation of psychedelics bound to 5-HT2A and other 5-HT receptors provides a glimpse into their molecular actions. Combined with structure-activity relationship studies and comparative analysis with nonpsychedelic 5-HT receptor ligands, these studies will undoubtedly facilitate the design of novel probes to investigate the different facets of psychedelic function *in vivo*.

### Implications for drug pharmacology and development of therapies

Overall, the reported structures of psychedelic-bound receptors have uncovered key aspects of psychedelic pharmacology. For instance, structural and functional work illuminated the molecular mechanisms underlying LSD’s slow receptor dissociation kinetics, which were suggested to play a role in the drug’s prolonged actions *in vivo*. Moreover, the same studies showed that LSD’s long receptor residence time is critical for potent and efficacious β-arrestin recruitment to the 5-HT2A receptor (116). Of note, 5-HT2A–mediated β-arrestin recruitment was shown to play a key role in LSD’s psychedelic effects in rodent models (115, 116). While these are intriguing findings, discovery of 5-HT2A ligands with divergent pharmacological activities and signaling preferences (118, 119, 120) indicate that the link between β-arrestin recruitment activity and psychedelic effects warrants further investigation.

More broadly, it is currently unknown how some 5-HT2A agonists such as LSD, psilocin, or mescaline are hallucinogenic, while others including 5-HT and lisuride are not. The pharmacological effects of the LSD congener lisuride are particularly puzzling, as both appear to be potent partial agonists at 5-HT2A. Structural studies of lisuride bound to 5-HT2B (126) and 5-HT2A (118), however, have uncovered a unique binding pose of the compound when compared to its LSD counterpart. Despite their chemical similarities, lisuride and LSD possess opposing stereochemical configurations with respect to their different ergoline substituents, resulting in distinct interactions with residues in the extended binding pockets of both receptors (Fig. 4, *A* and *D*). Although lisuride and LSD exhibit rather subtle differences in their 5-HT2A signaling profiles (118), lisuride is in fact an antagonist at 5-HT2B, whereas LSD is an agonist (126). This finding implies the possibility that modulatory effects, exerted by receptors other than 5-HT2A, can profoundly affect psychedelic pharmacology. While 5-HT2A appears to be the key mediator of psychedelic effects, studies have already uncovered important modulatory roles of other 5-HT receptors, such as 5-HT1A and 5-HT2C (112, 113).

Novel tool compounds are thus imperative to explore the precise 5-HT2A–mediated signaling pathways and the role of other receptors that lead to different aspects of the psychedelic experience. Several parallel approaches have leveraged existing structural data to develop chemically novel 5-HT2A agonists with distinct pharmacological profiles. For instance, virtual ligand screening approaches led to a novel 5-HT2A agonist scaffold seemingly devoid of hallucinogenic properties in animal models (Fig. 4*C*) (120). In a parallel study, structure-guided rational design similarly led to a nonhallucinogenic 5-HT2A agonist (118). These and other novel 5-HT2A agonists (119) display a range of signaling bias profiles different from that of known psychedelics, highlighting their usefulness in elucidating the key signaling pathways involved in the many dimensions of the psychedelic experience in future work. Psychedelics do not pose significant physical harm to the user, and existing antipsychotics and anxiolytics have proven successful in managing the psychoactive effects of an acute “overdose” (7), providing an additional measure for safe usage. Due to the promising outlook of psychedelic drugs in the treatment of several neuropsychiatric disorders, such as substance use disorders and depression, much effort has been dedicated to developing even safer psychedelic-derived medicines that lack the hallucinogenic effects. However, it remains to be seen if the therapeutic and hallucinogenic effects can be separated, since users ascribe much of the therapeutic value of these drugs to the psychedelic experience as a whole (7).

## Conclusions

We herein summarize recent structural studies that elucidate how drugs of abuse, such as opioids, cannabinoids, and psychedelics, bind to and activate their respective GPCR targets. These timely studies provide a valuable molecular context to previous pharmacological work. They further provide a structural framework to better understand the potential of cannabinoids and psychedelics in the treatment of various neuropsychiatric disorders and the consequences of the continuing application of opioids in pain management. Not only does the recent structural work elucidate key pharmacological aspects of drug signaling but it also directly facilitates structure-based efforts toward the generation of novel chemical probes (136, 137). Future studies deploying these tools are poised to uncover additional aspects of drug pharmacology, and, hopefully, facilitate the development of these probes into candidates for clinical trials.

Unfortunately, understanding ligand molecular recognition at GPCRs using structural biology approaches is only one part of the challenge of designing new chemical probes or drug candidates. The other challenge lies in designing pathway-selective chemical tools, but structural information into binding modes necessary for biased agonism is severely lacking. Biased chemical probes will be highly valuable in dissecting which GPCR signaling pathways are necessary for clinically relevant outcomes, and which may lead to adverse effects. For example, much progress has been made in designing seemingly nonpsychedelic 5-HT2A agonists that are devoid of hallucinogenic activity in rodent models, while retaining antidepressant and/or antiaddiction potential. However, which signaling pathways are necessary for therapeutic *versus* side effects for each respective GPCR (opioid, cannabinoid, serotonin) is still under investigation.

While targeting their corresponding GPCRs represents a direct way to modulate or mitigate the effects of opioids, cannabis, and psychedelics, there are many understudied GPCRs in modulatory circuitry that could be targeted for treating substance abuse and other drug-related maladies. For instance, synthetic partial and full agonists of the trace amine-associated receptor 1, which is activated by amphetamines (138), are effective as methamphetamine replacement therapy in preclinical studies (139, 140). Other examples include GPR6, an orphan GPCR closely related to cannabinoid receptors, whose striatal expression, regulation of dopamine signaling, and response to cannabinoids, make it a potential target for the treatment of drug addiction (141, 142). Another potential target is GPR151, an orphan GPCR expressed in the medial habenula that previously showed promise as a target for treating nicotine addiction (143, 144). Lastly, a recent genome-wide association study identified a correlation between a missense mutation of the striatally expressed orphan receptor GPR101 and cigarette consumption, offering another burgeoning opportunity for drug development (145).

The study of illicit drugs and the concomitant development of novel pharmacotherapies has two seemingly juxtaposed goals: (i) to combat their harmful effects, as is the case for opioids and (ii) to harness their therapeutic potential, as is the case for psychedelics. These goals need to contend with an ever-evolving landscape affected by their societal use and their associated legal framework. For instance, the current social perception of opioids is centered around their role in a deadly drug epidemic, rather than their use as critical tools to manage pain inside and outside the clinic. By contrast, cannabis, psilocybin, and ketamine are now hailed as potential solutions to seemingly all neuropsychiatric illnesses, rather than powerful psychoactive substances with profound short-term and long-term effects on human psychophysiology. Regardless of how the pendulum of social perception swings, the pharmacology of these compounds remains the same.

Overall, one thing is clear—a better understanding of the societal, physiological, pharmacological, and molecular effects of these drugs requires more research. In that respect, drug scheduling has done more harm than good by preventing critical avenues of research and perhaps even limiting access to what can now be considered as essential medicines (103). Investigating the molecular mechanisms of clearly highly efficacious drugs, as reviewed herein, can greatly facilitate the development of novel or improvement of existing medications—particularly in light of the high failure rate of clinical trials due to lack of efficacy.

## Conflict of interest

D. W. has consulted for Otsuka Pharmaceutical, Longboard Pharmaceuticals and Ocean Bio Ltd on the design of psychedelic-based therapeutics. None of the companies listed herein contributed to the funding or narrative of the manuscript. The other authors declare that they have no conflicts of interest with the contents of this article.
